# Brain magnetic resonance imaging of patients with spinal muscular atrophy type 2 and 3

**DOI:** 10.1016/j.nicl.2024.103708

**Published:** 2024-11-14

**Authors:** Marloes Stam, Harold H.G. Tan, Ruben Schmidt, Martijn P. van den Heuvel, Leonard H. van den Berg, Renske I. Wadman, W. Ludo van der Pol

**Affiliations:** a UMC Utrecht Brain Center, Department of Neurology, University Medical Center Utrecht, Utrecht University, Utrecht, the Netherlands; b Connectome Lab, Department of Complex Trait Genetics, Center for Neurogenomics and Cognitive Research, Amsterdam Neuroscience, Vrije Universiteit Amsterdam, Amsterdam, the Netherlands; c Department of Child Psychiatry, Amsterdam Neuroscience, Amsterdam UMC, Amsterdam, the Netherlands

**Keywords:** Spinal muscular atrophy, SMA, MRI, Brain, Cortical thickness, Thalamus

## Abstract

**Background and objective:**

Proximal spinal muscular atrophy (SMA) is caused by deficiency of the ubiquitously expressed survival motor neuron protein. Although primarily a hereditary lower motor neuron disease, it is probably also characterized by abnormalities in other organs. Brain abnormalities and cognitive impairment have been reported in severe SMA. We aimed to systematically investigate brain structure in SMA using MRI.

**Methods:**

We acquired high-resolution T1-weighted images of treatment-naive patients with SMA, age- and sex-matched healthy and disease controls with other neuromuscular diseases, on a 3 T MRI scanner. We performed vertex-wise whole brain analysis and region of interest analysis of cortical thickness (CT), and volumetric analysis of the thalamus and compared findings in patients and controls using multiple linear regression models and Wald test. We correlated structural abnormalities with motor function as assessed by the Hammersmith Functional Motor Scale Expanded (HFMSE) and SMA Functional Rating Scale (SMA-FRS).

**Results:**

We included 30 patients, 12–70 years old, with SMA type 2 and 3, 30 age- and sex-matched healthy controls and 17 disease controls (with distal SMA, hereditary motor and sensory neuropathy, multifocal motor neuropathy, progressive muscular atrophy and segmental SMA). We found a reduced CT in patients with SMA compared to healthy controls at the precentral, postcentral and medial orbitofrontal gyri and at the temporal pole (mean differences −0.059(p = 0.04); −0.055(p = 0.04), −0.06(p = 0.04); −0.17 mm(p = 0.001)). Differences at the precentral gyrus and temporal pole were most pronounced in SMA type 2 (mean differences −0.07(p = 0.045); −0.26 mm(p < 0.001)) and were also present compared to disease controls (mean differences −0.08(p = 0.048); −0.19 mm(p = 0.003)). There was a positive correlation between CT at the temporal pole with motor function. Compared to healthy controls, we found a reduced volume of the whole thalamus (mean difference −325 mm^3^(p = 0.03)) and of the anterior, ventral and intralaminar thalamic nuclei (mean differences −9.9(p = 0.02); −157(p = 0.01); −24.2 mm^3^(p = 0.02) in patients with SMA and a positive correlation between these volumes and motor function.

**Conclusion:**

MRI shows structural changes in motor and non-motor regions of the cortex and the thalamus of patients with SMA type 2 and 3, indicating that SMA pathology is not confined to motor neurons.

## Highlights

• MRI shows brain changes in patients with SMA in both motor and non-motor areas.
• Cortical thickness of several gyri is reduced in patients with SMA compared to healthy controls.
• Thalamic volume is reduced in SMA and correlates with motor function.

## 1 Introduction

Hereditary proximal spinal muscular atrophy (SMA) is characterized by lower motor neuron degeneration, progressive muscle weakness and atrophy and is caused by deficiency of the survival motor neuron (SMN) protein due to the homozygous loss-of-function of the *SMN1 gene* on chromosome 5q (Lefebvre et al., 1995, Wadman et al., 2018, Wijngaarde et al., 2020). The presence of a second human *SMN* gene (*SMN2*) ensures the production of residual levels of SMN protein, sufficient to sustain mechanisms vital for embryonal development but not for postnatal motor neuron function (Ramos et al., 2019). SMN protein is ubiquitously expressed, and its deficiency affects numerous basic cell biological processes (Singh et al., 2017). We have learned from animal and patient studies that motor neurons in the ventral horn of the spinal cord are most vulnerable to SMN protein deficiency, but that other organs and tissues also show anatomical, histological or physiological abnormalities (Martínez-Hernández et al., 2009, Wadman et al., 2012, Shababi et al., 2014, El Mendili et al., 2016, Stam et al., 2019, Habets et al., 2022). This includes pathological changes of the brain that have been observed *postmortem* in the most severe cases of SMA (Masson et al., 2021). *In vivo* brain structure and function in patients with SMA, particularly in the more chronic forms, have not been studied in detail, but mainly small-sized MRI studies of brains of patients with SMA have shown changes in the cerebellum, cerebral gray and white matter and changes in subcortical structures, including the thalamus (Querin et al., 2019, de Borba et al., 2020, Mugisha et al., 2023, Shen et al., 2023). Genetic SMN-augmenting therapies have dramatically improved survival and motor function in SMA. However, recent reports that documented impaired cognitive development of children with SMA type 1 following SMA-specific genetic treatment (Kölbel et al., 2024) highlight the importance of insights in the longer-term consequences of SMN depletion on development and pathology of the brain. To study brain anatomy in SMA systematically, we assessed changes in cortical thickness and thalamic volume using MRI in patients with SMA and healthy and disease controls.

## 2 Material and methods

We conducted this cross-sectional observational study at the Netherlands SMA Center of the University Medical Center Utrecht.

### 2.1 Participants

We recruited patients aged 12 years or older with a genetically confirmed, clinical diagnosis of SMA type 2 or 3 from the national SMA registry (Wadman et al., 2017). We defined SMA types according to the 1992 SMA consortium meeting definition; SMA type 2 with age of symptom onset between 6–18 months of age, and the ability to sit independently at any stage in life; SMA type 3 with symptom onset between 18 months and 30 years of age and the ability to walk independently at any point in life (Munsat and Davies, 1992, Zerres and Rudnik-Schöneborn, 1995). In case of discrepancy between age at symptom onset and highest achieved motor milestone, the latter was used to determine SMA type. We furthermore used the subdivision in type 3a and 3b, i.e. symptom onset before or after 3 years of age (Zerres & Rudnik-Schöneborn, 1995). Participants recruited healthy controls of approximately the same age through their network. We recruited disease controls with a confirmed diagnosis of neuromuscular disease but without central nervous system involvement through the pediatric and adult neuromuscular outpatient clinics of the University Medical Center Utrecht and one through the rehabilitation Center Rijndam, Rotterdam, The Netherlands. As additional controls, we used MRI scans of healthy and disease controls of two previous studies in our hospital that employed equivalent settings: One study was conducted at the ALS Center, Department of Neurology (Walhout et al., 2015) and the other at the Neuroimaging in Childhood (NICHE) Lab, Department of Psychiatry (Bos et al., 2017), both at the University Medical Center Utrecht. All 30 healthy controls and 17 disease controls were age- and sex-matched to the SMA patients (Salat et al., 2004, Hughes et al., 2012, Gennatas et al., 2017). Exclusion criteria were: any history or presence of brain injury, epilepsy, psychiatric illness and other cerebral disease; any intoxication or medication known to have an association with motor neuron dysfunction; any type of ventilation; pronounced swallowing disorders and/or risk of hypoventilation in a prolonged supine position (i.e. >15% postural change in forced vital capacity between sitting and lying supine or symptoms of nocturnal hypoventilation); (relative) contra-indications for 3 Tesla MRI such as claustrophobia, pregnancy and/or medical devices or metal implants such as spinal rods that are incompatible with MR Imaging.

### 2.2 Standard protocol approvals, registrations, and patient consents

The Medical Ethics Review Committee (MERC) of the University Medical Center Utrecht approved the study protocol. We obtained written informed consent from all participants and from parents or legal guardians of participants younger than 18 years. Healthy controls who had previously participated in a different study at our department had given written informed consent for the re-use of their data.

### 2.3 Clinical outcome measures

We determined *SMN2* copy numbers of patients with SMA using Multiplex Ligation-dependent Probe Amplification (MLPA) analysis (SALSA MLPA kit P060 version B2; https://www.mlpa.com; https://www.mrcholland.com) (Wadman et al., 2017). We used the Hammersmith Functional Motor Scale Expanded (HFMSE) to assess motor function (O’Hagen et al., 2007) and the SMA Functional Rating Scale (SMA-FRS) as a patient-reported functional scale. The latter is a functional scale modified from the Amyotrophic Lateral Sclerosis Functional Rating Scale (ALS-FRS) and reflects important aspects of daily living (ACTS phase I-II Study Group, 1996).

### 2.4 MRI protocol and data acquisition

We used the same 3 Tesla MR scanner (Achieva Medical Scanner, Philips), a SENSE 8-channel receiver head-coil and the same scan protocol for all participants to acquire high resolution T1 weighted images. Acquisition parameters are summarized in Table 1.

**Table 1:** MRI acquisition parameters.

**Sequence name**	**T1-weighted (3D-FFE)**
TE (ms)	4.6
TR (ms)	10
Flip angle	8°
Field of view (mm)	176 × 240 × 240
Acquisition matrix	304 × 299
Voxel size (mm)	0.8 × 0.75 × 0.75
Slice orientation	sagittal
Slices	220
Acquisition time (min)	11:02

*Abbreviations: MRI: magnetic resonance imaging; 3D-FFE: 3-dimensional fast field echo; TE: echo time; TR: repetition time*.

### 2.5 Data processing

We (pre)processed T1-weighted images with FreeSurfer image analysis suite (version 7.1.1; https://surfer.nmr.mgh.harvard.edu/), according to established FreeSurfer pipelines, including removal of non-brain tissue, determination of the boundary between white and gray matter and gray matter and the CSF (i.e. pial surface) to reconstruct the cortical layer. We computed surface-based vertex-wise measures of cortical thickness (Fischl B., 2012). The cortex was automatically parcellated into 34 gyral-based cortical regions per hemisphere based on the Desikan/Killiany Atlas (Desikan et al, 2006), allowing comparison of regional cortical differences. To investigate thalamic changes in SMA, we used the FreeSurfer thalamic nuclei segmentation tool that subdivides the thalamus into 25 separate nuclei (excluding the reticular nucleus, which largely consists of nerve fibres) clustered into six groups, based on anatomical location: i.e. anterior, lateral, ventral, intralaminar, medial and posterior groups (Iglesias et al., 2018). After FreeSurfer processing, we inspected image output visually for errors. When errors influencing measurement of cortical thickness or thalamic volume were present, we excluded the scan from the analysis. To reduce risk of bias, we did not correct errors manually.

### 2.6 Statistical analysis

We used R for statistical computing (R version 3.5.2). A p-value of <0.05 was statistically significant. Estimated marginal means were used to display the effect sizes and were computed using the ‘emmeans’ package. We did not correct for multiple comparisons because this study was exploratory. We used the mean cortical thickness and thalamic volumes of both hemispheres for analyses.

#### 2.6.1 Cortical thickness

We performed a whole-brain, region-wise analysis of mean cortical thickness using a multiple linear regression model adjusting for age and sex to compare between participant groups. The thirty-four cortical regions were defined by the Desikan/Killiany atlas (Desikan et al., 2006). We applied the Wald-test for p-value calculations, using a threshold of significance at p < 0.05.

#### 2.6.2 Subcortical structures: Volumetric analysis of the thalamus

We compared mean volume of the whole thalamus and mean volumes of thalamic nuclei between patients with SMA and controls. We grouped the nuclei into the six groups defined by FreeSurfer: 1) anterior group, including the anteroventral nucleus; 2) lateral group, including laterodorsal and lateral posterior nuclei; 3) ventral group, including ventral anterior, ventral anterior magnocellular, ventral lateral anterior, ventral lateral posterior, ventral posterolateral and ventromedial nuclei; 4) intralaminar group, including central medial, central lateral, paracentral, centromedian and parafascicular nuclei; 5) medial group, including paratenial, reuniens (medial ventral), mediodorsal medial magnocellular and mediodorsal lateral parvocellular nuclei; 6) posterior group, including lateral geniculate, medial geniculate, limitans (suprageniculate), pulvinar anterior, pulvinar medial, pulvinar lateral and pulvinar inferior nuclei (Iglesias et al., 2018). We used a multiple linear regression model, similar to the region-wise cortical thickness analysis, but included estimated intracranial volume as a variable to the model (Barnes et al., 2010).

In case of significant differences between SMA patients and healthy controls, we performed additional analyses to explore the contribution of SMA type and we assessed associations with HFMSE and SMA-FRS scores, using multiple linear regression. To investigate whether (sub)cortical anatomical changes were SMA-specific, we compared SMA patients and disease controls.

## 3 Results

### 3.1 Participants

We included 30 patients with SMA type 2 and 3 and 30 age- and sex-matched healthy controls. We enrolled 17 disease controls (1 distal SMA, 1 hereditary motor and sensory neuropathy (HMSN), 6 multifocal motor neuropathy (MMN), 8 progressive muscular atrophy (PMA), 1 segmental SMA). None of the patients with SMA were treated with SMN-targeted drugs, since these were not reimbursed at the time of this study. Participant characteristics are summarized in Table 2.

**Table 2:** Participant characteristics.

	**SMA**				**Controls**	
	**total**	**type 2**	**type 3a**	**type 3b**	**healthy**	**disease**
**N**	30	15	3	12	30	17
**Sex, male (%)**	12 (40)	6 (40)	1 (33)	5 (42)	13 (43)	13 (77)
**Age, years, mean (SD)**	36 (17)	30 (18)	42 (10)	41 (17)	36 (17)	49 (18)
**Handedness, right (%)**	23 (77)	12 (80)	2 (67)	9 (75)	27 (90)	16 (94)
**HFMSE, median (range)**	12 (0–66)	3 (0–24)	20 (12–30)	45 (3–66)	66 (−)	−
**SMA-FRS, median (range)**	13.5 (0–49)	7 (0–21)	31 (28–40)	44.5 (3–49)	50 (−)	−
**SMN2 copy number**						
**2**	2	−	1*	1**	−	−
**3**	14	12	1	1	−	−
**4**	14	3	1	10	−	−

*Abbreviations: SMA: spinal muscular atrophy; HFMSE: Hammersmith Motor Scale Expanded; SMA-FRS:*

*SMA Functional Rating Scale; SMN2: Survival Motor Neuron gene 2*.

*heterozygous deletion of SMN1 and a point mutation in exon 4 (c.542A > G) on the other allele, with 2.

SMN2 (Wadman et al., 2017).

**2SMN2 homzygous mutation in SMN2, c.859 G > C (Prior et al., 2009).

### 3.2 Data quality

None of the participants had to be excluded due to coincidental finding of structural abnormalities. Visual inspection of segmentation after FreeSurfer processing did not reveal segmentation errors that would interfere with measurements of cortical thickness or subcortical volumes. Therefore, we used all acquired MRI scans for analyses.

### 3.3 Cortical thickness

**Fig. 1:**
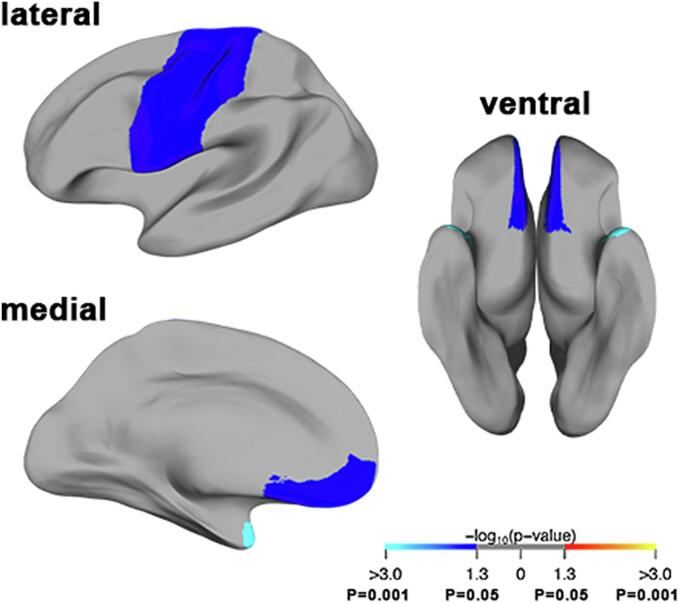
Regions with reduced mean cortical thickness in patients with SMA compared to healthy controls Results of whole brain, region-wise analysis of mean cortical thickness in patients with SMA compared to healthy controls. A blue value indicates that the cortex is significantly thinner in SMA patients compared to healthy controls: i.e. at the precentral, postcentral, medial orbitofrontal gyrus and temporal pole. The color bar shows the corresponding p-value*.*

**Fig. 2:**
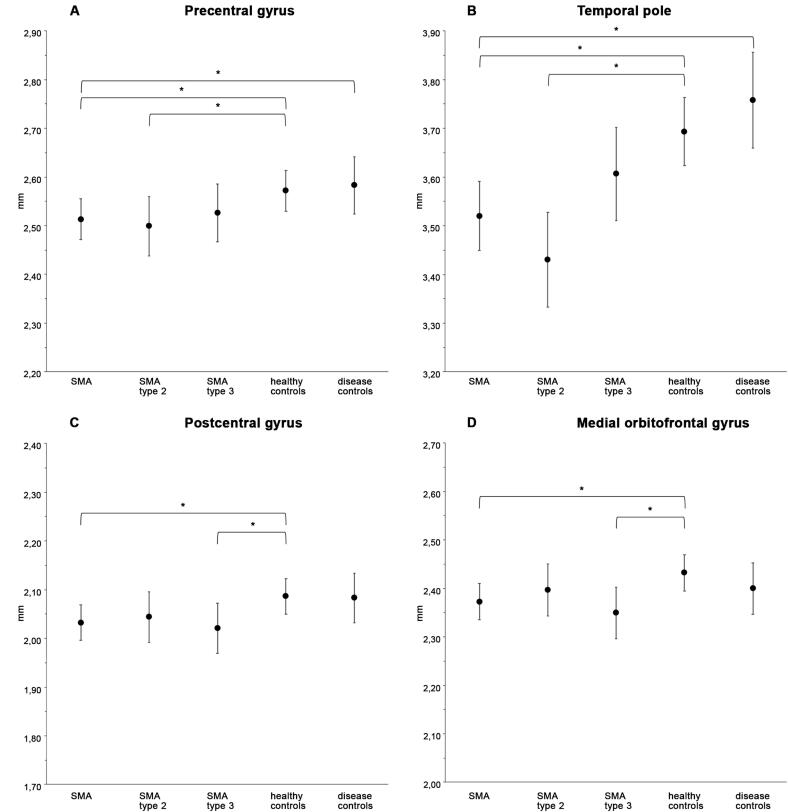
Regions with reduced mean cortical thickness in SMA patients compared to healthy controls, including subtype analyses. Results of participant subgroup analyses of mean cortical thickness. These were performed in brain regions that were found to be significantly different between patients with SMA and healthy controls in the whole-brain, region-wise analysis. Estimated marginal means with 95 % confidence intervals; *p < 0.05*.*

Region-wise analysis showed that mean cortical thickness in patients with SMA was significantly reduced compared to healthy controls at the precentral (mean difference −0.059 mm; p=0.038), postcentral (mean difference −0.055 mm; p=0.043) and medial orbitofrontal (mean difference −0.060; p=0.038) gyri and at the temporal pole (mean difference −0.174; p=0.001) (Fig. 1, Fig. 2 and Table 3). Subgroup analyses for these differences showed a reduced mean cortical thickness at the precentral gyrus and temporal pole in patients with SMA type 2 (mean difference −0.072 mm (p=0.045); −0.263 mm (p<0.001) resp.), but not in patients with SMA type 3, compared to healthy controls. At the postcentral and medial orbitofrontal gyri there was a reduced mean cortical thickness in patients with SMA type 3 (mean difference −0.067 mm (p=0.046); −0.089 mm (p=0.01) resp.), but not in patients with SMA type 2, compared to healthy controls. Compared to disease controls there was a reduced mean cortical thickness in patients with SMA at the precentral gyrus and temporal pole (mean difference −0.083 mm (p=0.048); −0.186 mm (p=0.003) resp.) Results are summarized in Fig. 2 and Table 3 and additional data are listed in eTable1.

**Table 3:** Summary of findings.

	**SMA patients**	**SMA type 2**	**SMA type 3**	**healthy controls**	**disease controls**	**comparisons◦**	**p-value^**	**correlation to clinical score (for SMA patients)**
	EMM, (95 %CI)*	EMM, (95 %CI)*	EMM, (95 %CI)*	EMM, (95 %CI)*	EMM, (95 %CI)*			*β (95 %CI; p-value)*
***cortical thickness (mm)***								
								
**precentral gyrus**	2.513 (2.471–2.555)	2.499 (2.438–2.559)	2.527 (2.467–2.586)	2.572 (2.530–2.613)	2.583 (2.524–2.642)	SMA vs HC	0.038	
						SMA vs DC	0.048	
						SMA 2 vs HC	0.045	
**postcentral gyrus**	2.032 (1.996–2.069)	2.044 (1.991–2.096)	2.021 (1.970–2.072)	2.086 (2.050–2.123)	2.083 (2.032–2.134)	SMA vs HC	0.043	
						SMA 3 vs HC	0.046	
**temporal pole**	3.520 (3.450–3.591)	3.430 (3.333–3.528)	3.606 (3.511–3.702)	3.693 (3.623–3.764)	3.758 (3.659–3.857)	SMA vs HC	0.001	SMA-FRS: 0.005 (0.002–0.009; 0.002) HFMSE: 0.004 (0.002–0.007; 0.003)
						SMA vs DC	0.003	
						SMA 2 vs HC	<0.001	
**medial orbitofrontal gyrus**	2.373 (2.335–2.410)	2.397 (2.343–2.451)	2.349 (2.296–2.402)	2.432 (2.394–2.470)	2.400 (2.347–2.453)	SMA vs HC	0.038	
						SMA 3 vs HC	0.01	
***thalamic volumes (mm3)***								
**whole thalamus**	7309 (7109–7510)	7076 (6797–7355)	7533 (7260–7807)	7635 (7432–7838)	7294 (7008–7580)	SMA vs HC	0.032	SMA-FRS: 13.83 (5.12–22.54; 0.003) HFMSE: 10.15 (3.00–17.30; 0.007)
						SMA 2 vs HC	0.006	
**anterior nuclei group**	141 (135–147)	139 (131–148)	143 (134–151)	151 (145–157)	142 (134–151)	SMA vs HC	0.02	
						SMA 2 vs HC	0.04	
**ventral nuclei group**	2821 (2737–2904)	2701 (2588–2815)	2936 (2824–3047)	2976 (2892–3060)	2853 (2734–2972)	SMA vs HC	0.01	SMA-FRS: 7.63 (4.41–10.84; <0.001) HFMSE: 5.66 (2.94–8.37; <0.001)
						SMA 2 vs HC	<0.001	
**intralaminar nuclei group**	422 (407–437)	401 (380–421)	442 (422–462)	446 (431–462)	431 (410–453)	SMA vs HC	0.02	SMA-FRS: 1.14 (0.46–1.91; 0.002) HFMSE: 0.84 (0.28–1.39; 0.005)
						SMA 2 vs HC	<0.001	

^p-values are not corrected for multiple comparisons.

*We report estimated marginal means and 95 %CI from a multiple linear regression model including all groups to show effect-sizes.

◦only comparisons between groups with significant differences are reported in this table.

Abbreviations: EMM = estimated marginal means; β = beta coefficient of regression model; SMA-FRS = SMA Functional Rating Scale; HFMSE = Hammersmith.

Functional Motor Scale Expanded, HC = healthy controls, DC = disease controls.

**Fig. 3:**
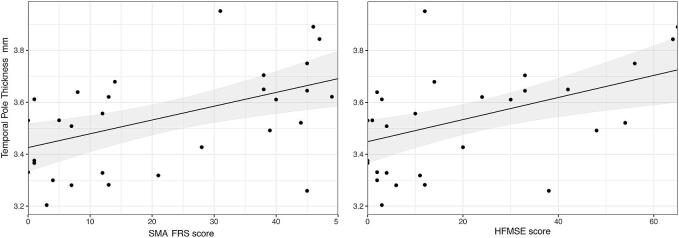
Correlation between cortical thickness at the temporal pole and clinical scores in SMA patients. Results of correlation analysis by multivariable linear regression model. There is a positive correlation between cortical thickness at the temporal pole and SMA-FRS score (β: 0.005; 95 %CI: 0.002–0.009; FDR corrected: p = 0,006) and HFMSE (β: 0.004; 95 %CI: 0.002–0.007; FDR corrected p-value = 0.006). Note that the individual data points are raw values, whereas the correlation lines are the result of the multivariable linear model. SMA-FRS = SMA Functional Rating Scale; HFMSE = Hammersmith Functional Motor Scale Expanded*.*

Analyses of correlation to clinical scores showed a positive correlation between cortical thickness at the temporal pole and SMA-FRS score (β: 0.005; 95%CI: 0.002–0.009; p=0.002; FDR corrected p=0.006) and HFMSE score (β: 0.004; 95%CI: 0.002–0.007; p=0.003; FDR corrected p=0.006) (Fig. 3).

### 3.4 Subcortical analysis: Volumetric analysis of the thalamus and its nuclei

#### 3.4.1 Whole thalamus volume

**Fig. 4:**
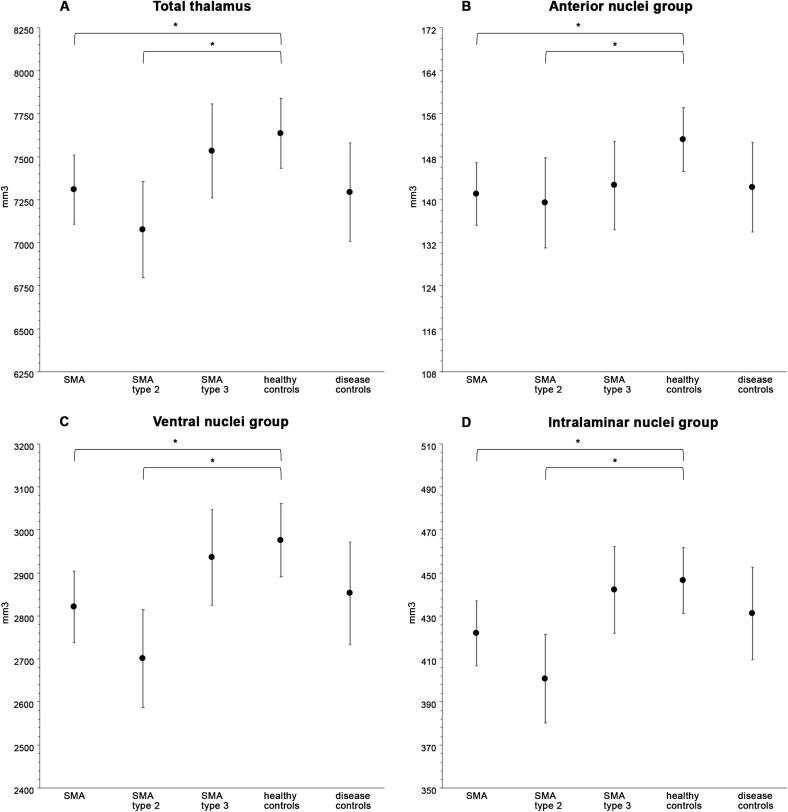
Reduced thalamic volumes in SMA patients compared to healthy controls, including subtype analyses. Results of participant subgroup analyses of thalamic volumes. These were performed for volumes/thalamic nuclei that were found to be significantly different between patients with SMA and healthy controls in the initial analysis: i.e. the whole thalamus and anterior, ventral and intralaminar thalamic nuclei. Estimated marginal means with 95 % confidence intervals; *p < 0.05*.*

**Fig. 5:**
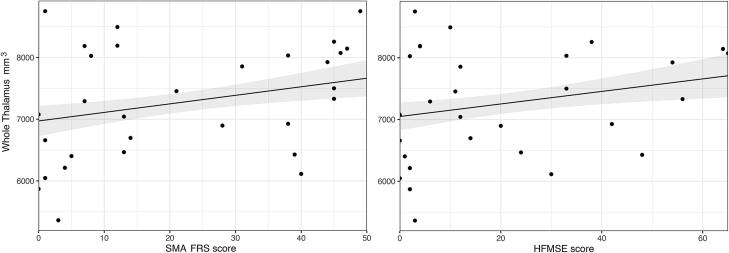
Correlation between thalamic volume and clinical scores in SMA patients. Results of correlation analysis by multivariable linear regression model. There is a positive correlation between mean thalamus volume and SMA-FRS score (β: 13.83; 95 %CI: 5.12–22.54; FDR corrected p-value = 0.007) and HFMSE score (β: 10.15; 95 %CI: 3.00–17.30; FDR corrected p-value = 0.012. Note that the individual data points are raw values, whereas the correlation lines are the result of the multivariable linear model. SMA-FRS = SMA Functional Rating Scale; HFMSE = Hammersmith Functional Motor Scale Expanded*.*

We found a reduced volume of the thalamus in patients with SMA compared to healthy controls (mean difference −325.3 mm^3^; p=0.032). Subgroup analyses showed a reduced volume in patients with SMA type 2 (mean difference −527.3 mm^3^; p=0.006), but not in patients with SMA type 3, compared to healthy controls. There was no significant difference between patients with SMA and disease controls (Fig. 4, Table 3 and eTable 2). Analyses of correlation to clinical scores showed a positive correlation between mean thalamus volume and SMA-FRS score (β: 13.83; 95%CI: 5.12–22.54; p=0.003; FDR corrected p=0.007) and HFMSE score (β: 10.15; 95%CI: 3.00–17.30; p=0.007; FDR corrected p=0.012) (Fig. 5).

#### 3.4.2 Thalamic nuclei

**Fig. 6:**
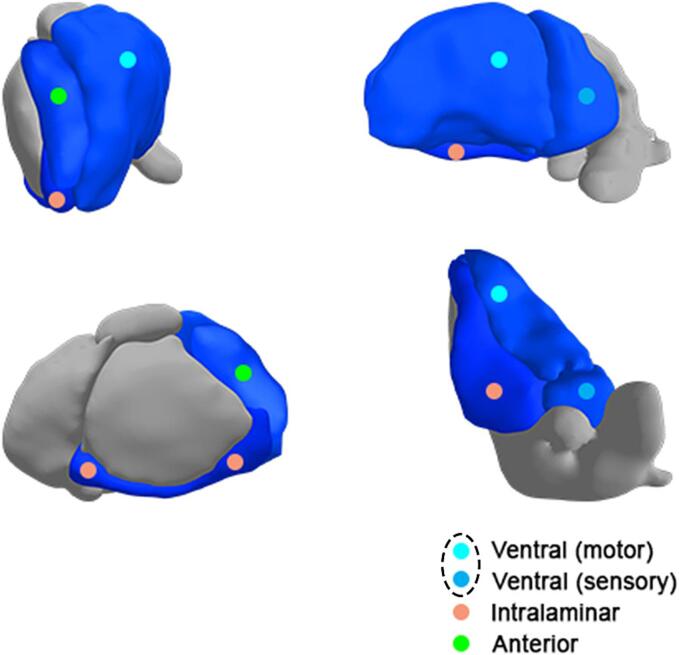
Thalamic nuclei with reduced volume in patients with SMA compared to healthy controls. Results of the analysis of volumes of thalamic nuclei in patients with SMA compared to healthy controls. Blue value indicates that the volume is significantly smaller in SMA patients compared to healthy controls (p-values ≤ 0.02), i.e. the anterior, ventral and intralaminar thalamic nuclei. Dashed circle: We analyzed the motor and sensory part of the ventral nucleus combined.

We found significantly smaller anterior, ventral and intralaminar thalamic nuclei in patients with SMA compared to healthy controls (mean difference −9.9 mm^3^ (p=0.02); −156.7 mm^3^ (p=0.01); −24.2 mm^3^ (p=0.02), resp.) (Fig. 4, Fig. 6, Table 3 and eTable2).

**Fig. 7:**
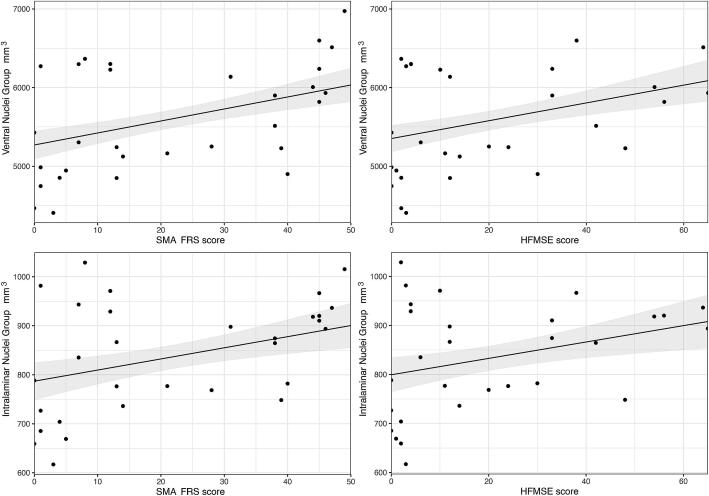
Correlation between volumes of thalamic nuclei and clinical scores in SMA patients. Results of correlation analysis by multivariable linear regression model. There is a positive correlation between ventral nuclei group volume and SMA-FRS score (β: 7.63; 95 %CI: 4.41–10.84; FDR corrected p-value < 0.001) and HFMSE score (β: 5.66; 95 %CI: 2.94–8.37; FDR corrected p-value = 0.001). There is a positive correlation between intralaminar nuclei group volume and SMA-FRS score (β:1.14; 95 %CI: 0.46–1.91; FDR corrected p-value = 0.006) and HFMSE score (β:0.84; 95 %CI: 0.28–1.39; FDR corrected p-value = 0.009) Note that the individual data points are raw values, whereas the correlation lines are the result of the multivariable linear model. SMA-FRS = SMA Functional Rating Scale; HFMSE = Hammersmith Functional Motor Scale Expanded*.*

Subgroup analysis for these differences showed a smaller volume of the anterior, ventral and intralaminar nuclei in patients with SMA type 2 (mean difference −11.1 mm^3^ (p=0.04); −259.5 mm^3^ (p<0.001); −42.3 mm^3^ (p<0.001), resp.), but not in patients with SMA type 3, compared to healthy controls. There were no significant differences between patients with SMA and disease controls (Fig. 4, Table 3 and eTable 2). Analyses of correlation to clinical scores showed a positive correlation between ventral nuclei group volume and SMA-FRS score (β: 7.63; 95%CI: 4.41–10.84; p<0.001; FDR corrected p<0.001) and HFMSE score (β: 5.66; 95%CI: 2.94–8.37; p<0.001; FDR corrected p=0.001), and between intralaminar nuclei group volume and SMA-FRS score (β: 1.14; 95%CI: 0.46–1.91; p=0.002; FDR corrected p=0.006) and HFMSE score (β: 0.84; 95%CI: 0.28–1.39; p=0.005; FDR corrected p=0.009) (Fig. 7).

## 4 Discussion

This neuroimaging study shows that patients with SMA display cerebral abnormalities in motor and non-motor regions compared to healthy age- and sex-matched controls. We found marked changes in the motor system, in particular thinning of the primary motor cortex, i.e. the precentral gyrus, and prominent reduced mean volume of the thalamus and in particular the ventral nuclei that harbor motor nuclei (Crossman and Neary, 2005). Changes were, however, more wide-spread and not limited to the motor system. Furthermore, changes were more pronounced in SMA type 2 than in type 3, suggesting a possible relation with disease severity. An important part of our findings is SMA-specific (i.e. not present in disease controls) and this indicates that not only the peripheral but also the central nervous system is affected in patients with SMA.

In previous studies using SMA mouse models and in case reports of post-mortem examination of SMA patients, cortical neuronal degeneration, loss of Betz cells in the motor cortex and thalamic lesions and degeneration have been found (Araki et al., 2003, Harding et al., 2015, Masson et al., 2021). Most of the data from human studies were obtained from severely affected patients with type 1, or even with a prenatal onset of symptoms (type 0). The reported abnormalities in brains may therefore reflect late or end-stage stage disease phenomena that might be the direct result of SMA-pathophysiology but could also be due to hypoxia secondary to respiratory failure. In this study the application of MRI in living patients shows that cerebral changes are also present in patients with chronic forms of SMA.

We are aware of three prior MRI brain studies in SMA (Querin et al., 2019, de Borba et al., 2020, Shen et al., 2023). One study focused on the cerebellum and reported reduced volumes of specific cerebellar lobules and cerebellar gray matter atrophy (de Borba et al., 2020). Our MRI scanning protocol focused on the hemispheres and therefore did not include the cerebellum. In the second study, the authors used a multi-modal MR imaging study of the brain and spinal cord in later onset SMA, i.e. types 3 (n=19) and 4 (n=6), and voxel-based analysis that revealed increased gray matter density in the precentral, dorsolateral prefrontal, orbitofrontal and anterior cingulate gyri in SMA patients compared to healthy controls. In addition, the authors reported an increased cortical volume of the left motor cortex of patients with type 4, which correlated with disease duration, as compared to type 3. No significant differences were found in cortical thickness (Querin et al., 2019). A third study reported alterations in cortex and subcortical structures in children with SMA type 2 (n=22) and 3 (n=21). Differences between patients with SMA and healthy controls were found in both motor and non-motor areas of the brain and included reduced white matter volumes in patients with SMA, reduced cortical volumes in some areas (precentral and central sulcus) but increased volumes in other areas (e.g. bilateral middle temporal). Interestingly but in contrast to our findings, cortical thickness was increased in patients with SMA throughout the entire brain. The study also reported heterogeneous alterations in subcortical volumes, i.e. an increase of the right hippocampus and bilateral pallidum in SMA, but reduced volumes of the corpus callosum (Shen et al., 2023).

In contrast to these few existing studies, we focused on a limited set of outcome measures, i.e. cortical thickness and thalamic volumes, based on prior findings from pathological studies (Araki et al., 2003, Harding et al., 2015, Masson et al., 2021) and to limit the numbers of statistical testing. In addition, we investigated correlations of these measures with disease severity using two clinical scores (HFMSE and SMA-FRS), rather than disease duration as a surrogate marker, which has previously been used. We found abnormalities in the brain of patients with SMA in both motor and non-motor areas, indicating that SMA pathology extends beyond the motor areas in the nervous system, including alterations in areas involved in cognitive functions, such as the temporal pole. Importantly, we found the most pronounced reduction of CT in patients with SMA type 2, suggesting a possible relation with disease severity. This may explain the reported lack of significant differences in patients with SMA (late onset) types 3 and 4 (Querin et al., 2019). However, findings in patients with SMA type 2 and 3 in the study by Shen et al. (Shen et al., 2023) were markedly different from ours; in contrast to the more pronounced thinning of CT and reduced thalamic volumes we found, Shen et al. (2024) report striking variation (i.e. both increased and decreased) in CT and subcortical volumes, which cannot be explained by the age difference. The authors suggested a combination of degenerative and compensatory mechanisms in reaction to SMN protein deficiency. However, we cannot exclude that methodological differences underlie discrepancies between the studies, such as the use of different regions-of interest and morphological outcome measures. For example, gray matter density and cortical thickness are non-equivalent and show different age and sex effects (Gennatas et al., 2017). We did not find a significant correlation between cortical thickness at the precentral gyrus (i.e. primary motor cortex) and motor scale scores. This may partly be due to the relatively small sample size and heterogeneity in remaining motor function, since subgroup analysis suggested lower CT values among patients with SMA type 2. Protocols for future studies should allow to investigate CT and study correlations of motor function scales with specific areas of the precentral gyrus, since muscle groups are differentially affected in SMA (e.g. lower limbs are affected earlier and more severely compared to upper limbs.(Deymeer et al., 1997, Piepers et al., 2008, Wadman et al., 2018) Finally, other measures such as cortical volume, gray matter density and cortical surface area should be further explored to investigate which imaging measure for cortical involvement is most sensitive.

SMN protein expression is relatively high throughout the brain and spinal cord and cerebral function may therefore be affected in patients with SMA. There are indeed recent concerns regarding cognition in symptomatic patients with SMA type 1 who received genetic treatment (Ngawa et al., 2023, Kölbel et al., 2024), but literature on cognition in SMA type 2 and 3 is scarce and showed conflicting results. Findings of a few recent studies suggest that SMA patients may perform worse than healthy controls on specific cognitive domains, including working memory, perceptual reasoning, visuospatial and executive function. Moreover, this may be related to disease severity (Polido et al., 2019, Kizina et al., 2021, Lenzoni et al., 2022). Obviously, we do not know whether the observed brain alterations are associated with cognition, but we hypothesize that our findings indicate that structural changes may also underlie non-motor symptoms in SMA. We think that combined brain imaging-cognition studies of patients with SMA are needed to validate this hypothesis. Whether the novel *SMN-*augmenting therapies improve non-motor symptoms to the same extent as weakness remains to be answered (Reilly et al., 2023). Importantly, we found the most pronounced structural changes in patients with SMA type 2. In addition, the correlations between reduced cortical thickness of certain brain areas and reduced thalamic (nuclei) volumes with the SMA-FRS and HFMSE clinical scores, could point towards a relation between severity of motor dysfunction and brain alterations.

This study obviously has its limitations. Although our sample size is appropriate for a neuroimaging study, there may still be limited power to detect smaller but relevant changes. Our experience was that the inclusion of disease controls was a significant hurdle, since the personal interest to participate was very small. We were able to increase the number of disease controls to an adequate number by including MRI-scans that were previously acquired in our department, but these participants did not perform the clinical outcome measures that we used in this study (i.e. SMA-FRS and HFMSE). More in general, it remains challenging to find appropriate disease controls for SMA, i.e. patients with peripheral nervous system abnormalities only, as with ongoing research, brain changes are found in a growing number of neuromuscular diseases (Angelini & Pinzan, 2019). Based on our findings of brain changes in non-motor areas in SMA, we would recommend to asses non-motor functions, such as cognition, in future SMA studies.

## 5 Conclusion

Cortical thinning of motor and non-motor gyri and reduced thalamic volumes are characteristics of patients with SMA. We observed a positive correlation between motor function scores and reduced thalamic volume and temporal pole cortical thickness. Abnormalities were more pronounced in SMA type 2 than type 3. Longitudinal studies could address the relation of structural brain changes and motor function, cognition and other non-motor functions and the effects of treatment.

## CRediT authorship contribution statement

**Marloes Stam:** Writing – review & editing, Writing – original draft, Visualization, Project administration, Methodology, Investigation, Formal analysis, Data curation, Conceptualization. **Harold H.G. Tan:** Writing – review & editing, Visualization, Formal analysis. **Ruben Schmidt:** Writing – review & editing, Software, Methodology, Conceptualization. **Martijn P. van den Heuvel:** Writing – review & editing, Software, Methodology, Conceptualization. **Leonard H. van den Berg:** Writing – review & editing, Supervision, Resources, Conceptualization. **Renske I. Wadman:** Writing – review & editing, Methodology, Conceptualization. **W. Ludo van der Pol:** Writing – review & editing, Writing – original draft, Supervision, Resources, Methodology, Funding acquisition, Formal analysis, Conceptualization.

## Funding

This study was supported by grants from the Prinses Beatrix Spierfonds (WAR13-07) and Stichting Spieren voor Spieren. Prinses Beatrix Spierfonds and Stichting Spieren voor Spieren had no role in study design, data collection, data analysis or interpretation, writing the report or decisions concerning submitting this paper.

## Declaration of Competing Interest

The authors declare the following financial interests/personal relationships which may be considered as potential competing interests: [Martijn P. van den Heuvel works as a consultant for ROCHE and is part of the editorial board of Human Brain Mapping. Leonard H. van den Berg serves on scientific advisory boards for the Prinses Beatrix Spierfonds and receives research support from the Prinses Beatrix Fonds. W. Ludo van der Pol served as an ad hoc member of scientific advisory boards (fee for service to employer) for Biogen, Biohaven, NMD Pharma, Scholar Rock, Roche and Novartis Genetherapies and receives research support from the Prinses Beatrix Spierfonds, EU Horizon 2020, Vriendenloterij and Stichting Spieren voor Spieren. Marloes Stam, Harold H. G. Tan, Ruben Schmidt and Renske I. Wadman report no competing interests].

## Data Availability

Data will be made available on request.

## References

[b0005] The ALS CNTF treatment study (ACTS) phase I-II Study Group. The Amyotrophic Lateral Sclerosis Functional Rating Scale. Assessment of activities of daily living in patients with amyotrophic lateral sclerosis. Arch Neurol. 1996; 53:141–147. 8639063

[b0010] Angelini C and Pinzan E. Advances in imaging of brain abnormalities in neuromuscular disease. Ther. Adv. Neurol. Disord. 2019; 12: 1756286419845567. Epub 2019 May 6. doi:10.1177/1756286419845567. 10.1177/1756286419845567 PMC6503605 31105770

[b0015] Araki S., Hayashi M., Tamagawa K. (2003). Neuropathological analysis in spinal muscular atrophy type II. *Acta Neuropathol.*.

[b0020] Barnes J., Ridgway G.R., Bartlett J. (2010). Head size, age and gender adjustment in MRI studies: a necessary nuisance?. *Neuroimage*.

[b0025] Bos D.J., Oranje B., Achterberg M. (2017). Structural and functional connectivity in children and adolescents with and without attention deficit/hyperactivity disorder. *J Child Psychol. Psychiatry*.

[b0030] **Crossman AR,** Neary D. Thalamus. In: Crossman AR, Neary D, eds. Neuroanatomy. Elsevier Churchill Livingstone 2005:123-128.

[b0035] de Borba F.C., Querin G., França M.C., Pradat P.F. (2020). Cerebellar degeneration in adult spinal muscular atrophy patients. *J. Neurol.*.

[b0040] Desikan R.S., Ségonne F., Fischl B. (2006). An automated labeling system for subdividing the human cerebral cortex on MRI scans into gyral based regions of interest. *Neuroimage*.

[b0045] Deymeer F., Serdaroglu P., Poda M., Gulsen-Parman Y., Ozcelik T., Ozdemir C. (1997). Segmental distribution of muscle weakness in SMA III: implications for deterioration in muscle strength with time. *Neuromuscul. Disord.*.

[b0050] El Mendili M.M., Lenglet T., Stojkovic T. (2016). Cervical spinal cord atrophy profile in adult SMN1-linked SMA. *PLoS One*.

[b0055] FreeSurfer F.B. (2012). *Neuroimage*.

[b0060] Gennatas E.D., Avants B.B., Wolf D.H. (2017). Age-related effects and sex differences in gray matter density, volume, mass and cortical thickness form childhood to young adulthood. *J. Neurosci.*.

[b0065] Habets L.E., Bartels B., Asselman F. (2022). Magnetic resonance reveals mitochondrial dysfunction and muscle remodelling in spinal muscular atrophy. *Brain*.

[b0070] Harding B.N., Kariya S., Monani U.R. (2015). Spectrum of neuropathophysiology in spinal muscular atrophy type I. *J. Neuropathol. Exp. Neurol.*.

[b0075] Hughes E.J., Bond J., Svrckova P. (2012). Regional changes in thalamic shape and volume with increasing age. *Neuroimage*.

[b0080] Iglesias J.E., Insausti R., Lerma-Usabiaga G. (2018). A probabilistic atlas of the human thalamic nuclei combining ex vivo MRI and histology. *Neuroimage*.

[b0085] Kizina K., Akkaya Y., Jokisch D. (2021). Cognitive Impairment in Adult Patients with 5q-Associated Spinal Muscular Atrophy. *Brain Sci.*.

[b0090] Kölbel H., Kopka M., Modler L. (2024). Impaired Neurodevelopment in Children with 5q-SMA - 2 Years After Newborn Screening. *J. Neuromuscul. Dis.*.

[b0095] Lefebvre S., Bürglen L., Reboullet S. (1995). Identification and characterization of a spinal muscular atrophy-determining gene. *Cell*.

[b0100] Lenzoni S., Semenza C., Calligaro D. (2022). Cognitive profiles and clinical factors in type III spinal muscular atrophy: a preliminary study. *Neuromuscul. Disord.*.

[b0105] Martínez-Hernández R., Soler-Botija C., Also E. (2009). The developmental pattern of myotubes in spinal muscular atrophy indicates prenatal delay of muscle maturation. *J. Neuropathol. Exp. Neurol.*.

[b0110] Masson R., Brusa C., Scoto M., Baranello G. (2021). Brain, cognition, and language development in spinal muscular atrophy type 1: a scoping review. *Dev. Med. Child Neurol.*..

[b0115] Mugisha N., Oliveira-Carneiro A., Behlim T., Oskoui M. (2023). Brain Magnetic Resonance Imaging (MRI) in spinal muscular atrophy: a scoping review. J. Neuromuscul. Dis*.*.

[b0120] Munsat T.L., Davies K.E. (1992). International SMA Consortium Meeting (26-28 June 1992, Bonn, Germany). *Neuromuscul. Disord.*.

[b0125] **Ngawa M,** Dal Farra F, Marinescu AD, Servais L. Longitudinal developmental profile of newborns and toddlers treated for spinal muscular atrophy. *Ther Adv Neurol Disord.* 2023;16:1-9, epub. doi:10.1177/17562864231154335. 10.1177/17562864231154335 PMC9944336 36846472

[b0130] O’Hagen J.M., Glanzman A.M., McDermott M.P. (2007). An expanded version of the Hammersmith Functional Motor Scale for SMA II and III patients. *Neuromuscul. Disord.*.

[b0135] Piepers S., van den Berg L.H., Brugman F. (2008). A natural history study of late onset spinal muscular atrophy types 3b and 4. *J. Neurol.*.

[b0140] Polido G.J., Miranda de M.M.V., Carvas N. (2019). Cognitive performance of children with spinal muscular atrophy: A systematic review. *Dement Neuropsychol.*.

[b0145] Prior T.W., Krainer A.R., Hua Y. (2009). A positive modifier of spinal muscular atrophy in the SMN2 gene. *Am. J. Hum. Genet.*.

[b0150] Querin G., El Mendili M.M., Lenglet T. (2019). The spinal and cerebral profile of adult spinal-muscular atrophy: a multimodal imaging study. *Neuroimage Clin*.

[b0155] Ramos D.M., d’Ydewalle C., Gabbeta V. (2019). Age-dependent SMN expression in disease-relevant tissue and implications for SMA treatment. *J. Clin. Invest.*.

[b0160] Reilly A., Chehade L., Kothary R. (2023). Curing SMA: are we there yet?. *Gene Ther.*.

[b0165] Salat D.H., Buckner R.L., Snyder A.Z. (2004). Thinning of the cerebral cortex in aging. *Cereb. Cortex*.

[b0170] Shababi M., Lorson C.L., Rudnik-Schöneborn S.S. (2014). Spinal muscular atrophy: a motor neuron disorder or a multi-organ disease?. *J. Anat.*.

[b0175] **Shen W,** Yan Z, Su S, et al. Gray and white matter abnormalities in children with type 2 and 3 SMA: A morphological assessment. *Eur. J. Pediart.* 2023 Dec 18; Doi: 10.1007/s00431-023-05397-z. Epub 2024 Jan 2. 10.1007/s00431-023-05397-z 38165463

[b0180] Singh R.N., Howell M.D., Ottesen E.W., Singh N.N. (2017). Diverse role of Survival Motor Neuron Protein. *Biochim. Biophys. Acta*.

[b0185] Stam M., Haakma W., Kuster L. (2019). Magnetic resonance imaging of the cervical spinal cord in spinal muscular atrophy. *Neuroimage Clin*.

[b0190] Wadman R.I., Vrancken A.F., van den Berg L.H., van der Pol W.L. (2012). Dysfunction of the neuromuscular junction in spinal muscular atrophy types 2 and 3. *Neurology*.

[b0195] Wadman R.I., Stam M., Gijzen M. (2017). Association of motor milestones, SMN2 copy and outcome in spinal muscular atrophy types 0-4. *J. Neurol. Neurosurg. Psychiatry*.

[b0200] Wadman R.I., Wijngaarde C.A., Stam M. (2018). Muscle strength and motor function throughout life in a cross-sectional cohort of 180 patients with spinal muscular atrophy types 1c–4. *Eur. J. Neurol.*.

[b0205] Walhout R., Westeneng H.J., Verstraete E. (2015). Cortical thickness in ALS: towards a marker for upper motor neuron involvement. *J. Neurol. Neurosurg. Psychiatry*.

[b0210] Wijngaarde C.A., Stam M., Otto L.A.M. (2020). Muscle strength and motor function in adolescents and adults with spinal muscular atrophy. *Neurology*.

[b0215] Zerres K., Rudnik-Schöneborn S. (1995). Natural History in Proximal Spinal Muscular Atrophy: Clinical Analysis of 445 Patients and Suggestions for a Modification of Existing Classifications. *Arch. Neurol.*.

