# Supplementary material for: Brain magnetic resonance imaging of patients with spinal muscular atrophy type 2 and 3
Source: Neuroimage Clin. 2024 Nov 14;44:103708. doi: 10.1016/j.nicl.2024.103708 (PMC11617753; doi:10.1016/j.nicl.2024.103708)
Supplement: Supplementary Data 1 [file mmc1.docx]

**Supplemental material**

To Research Article:

Magnetic resonance imaging of the brains of patients with spinal muscular atrophy

| **eTable 1.** |  |  |  |  |  |  |
| --- | --- | --- | --- | --- | --- | --- |
| **Regions with reduced cortical thickness in patients with SMA compared to healthy controls, including subgroup analyses. Results of multiple linear regression model, adjusted for age and sex** | | | | | | |
|  |  |  |  |  |  |  |
| **cortical region** | **mean (mm)** | **95%CI**  **(mm)** | **mean (mm)** | **95%CI**  **(mm)** | **mean difference (mm)** | **p-value*** |
|  | *Main analysis: patients with SMA vs healthy controls* | | | | | |
|  | SMA | | healthy controls | |  |  |
| precentral gyrus | 2.527 | (2.487-2.566) | 2.586 | (2.546-2.625) | -0,059 | 0,038 |
| temporal pole | 3.531 | (3.457-3.604) | 3.705 | (3.631-3.778) | -0,174 | 0,001 |
| postcentral gyrus | 2.044 | (2.006-2.082) | 2.098 | (2.061-2.136) | -0,055 | 0,043 |
| medial orbitofrontal gyrus | 2.389 | (2.348-2.429) | 2.448 | (2.408-2.488) | -0,060 | 0,038 |
|  | *Subgroup analysis: patients with SMA type 2 vs healthy controls* | | | | | |
|  | SMA type 2 | | healthy controls | |  |  |
| precentral gyrus | 2.521 | (2.464-2.579) | 2.593 | (2.553-2.634) | -0,072 | 0,045 |
| temporal pole | 3.447 | (3.349-3.544) | 3.71 | (3.641-3.779) | -0,263 | <0,001 |
| postcentral gyrus | 2.059 | (2.004-2.114) | 2.106 | (2.068-2.145) | -0,047 | 0,161 |
| medial orbitofrontal gyrus | 2.42 | (2.359-2.481) | 2.459 | (2.416-2.502) | -0,039 | 0,294 |
|  | *Subgroup analysis: patients with SMA type 3 vs healthy controls* | | | | | |
|  | SMA type 3 | | healthy controls | |  |  |
| precentral gyrus | 2.529 | (2.473-2.585) | 2.579 | (2.54-2.619) | -0,050 | 0,149 |
| temporal pole | 3.611 | (3.501-3.72) | 3.693 | (3.616-3.77) | -0,082 | 0,223 |
| postcentral gyrus | 2.024 | (1.97-2.078) | 2.091 | (2.053-2.129) | -0,067 | 0,046 |
| medial orbitofrontal gyrus | 2.349 | (2.295-2.403) | 2.438 | (2.4-2.476) | -0,089 | 0,010 |
|  | *Subgroup analysis: patients with SMA vs disease controls* | | | | | |
|  | SMA | | disease controls | |  |  |
| precentral gyrus | 2.5 | (2.454-2.545) | 2.582 | (2.519-2.646) | -0,083 | 0,048 |
| temporal pole | 3.528 | (3.462-3.593) | 3.714 | (3.622-3.806) | -0,186 | 0,003 |
| postcentral gyrus | 2.025 | (1.988-2.063) | 2.072 | (2.02-2.124) | -0,047 | 0,170 |
| medial orbitofrontal gyrus | 2.363 | (2.327-2.399) | 2.384 | (2.333-2.435) | -0,021 | 0,516 |
| ** p-values are not corrected for multiple comparisons due to the exploratory nature of this study* | | | | | | |

**eTable 2.**

**Thalamus and thalamic nuclei with reduced volume in patients with SMA compared to healthy controls, including subgroup analyses.**

**Results of multiple linear regression model, adjusted for age, sex and estimated intracranial volume.**

| **volume** | **mean (mm3)** | **95%CI (mm3)** | **mean (mm3)** | **95%CI (mm3)** | **mean difference (mm3)** | **p-value*** |
| --- | --- | --- | --- | --- | --- | --- |
|  | *Main analysis: patients with SMA vs healthy controls* | | | | | |
|  | SMA | | healthy controls | |  |  |
| thalamus total | 7380 | (7170-7590) | 7710 | (7490-7920) | -325,30 | 0,032 |
| anterior nuclei group | 142.5 | (136.7-148.3) | 152.4 | (146.5-158.3) | -9,93 | 0,019 |
| ventral nuclei group | 2841.2 | (2755-2926) | 2997.9 | (2910-3085) | -156,74 | 0,012 |
| intralaminar nuclei group | 425.7 | (410.9-440.5) | 449.9 | (434.8-464.9) | -24,16 | 0,025 |
|  | *Subgroup analysis: patients with SMA type 2 vs healthy controls* | | | | | |
|  | SMA type 2 | | healthy controls | |  |  |
| thalamus total | 7250 | (6950-7550) | 7780 | (7560-7990) | -527,27 | 0,006 |
| anterior nuclei group | 142.7 | (134.1-151.3) | 153.8 | (147.7-160) | -11,15 | 0,040 |
| ventral nuclei group | 2758.8 | (2644-2873) | 3018.4 | (2936-3100) | -259,53 | <0,001 |
| intralaminar nuclei group | 411.8 | (392.5-431) | 454.1 | (440.2-467.9) | -42,29 | <0,001 |
|  | *Subgroup analysis: patients with SMA type 3 vs healthy controls* | | | | | |
|  | SMA type 3 | | healthy controls | |  |  |
| thalamus total | 7580 | (7270-7890) | 7700 | (7480-7930) | -119,77 | 0,529 |
| anterior nuclei group | 143.7 | (135.1-152.3) | 152.3 | (146.1-158.4) | -8,58 | 0,107 |
| ventral nuclei group | 2951.2 | (2823-3079) | 3003.7 | (2911-3095) | -52,44 | 0,505 |
| intralaminar nuclei group | 443.6 | (422.8-464.5) | 451.1 | (436.1-466.1) | -7,45 | 0,561 |
|  | *Subgroup analysis: patients with SMA vs disease controls* | | | | | |
|  | SMA | | disease controls | |  |  |
| thalamus total | 7170 | (6990-7350) | 7300 | (7050-7560) | -132,11 | 0,425 |
| anterior nuclei group | 138.5 | (132.8-144.3) | 142.6 | (134.4-150.7) | -4,03 | 0,444 |
| ventral nuclei group | 2769.7 | (2690-2848) | 2857.3 | (2745-2968) | -87,58 | 0,226 |
| intralaminar nuclei group | 412.1 | (397.1-427.2) | 434.7 | (413.4-456) | -22,58 | 0,104 |
| ** p-values are not corrected for multiple comparisons due to the exploratory nature of this study* | | | | | |  |
